# Clinical observation of dexmedetomidine nasal spray in the treatment of sleep disorders on the first night after undergoing maxillofacial surgery: a single-center double-blind randomized controlled study

**DOI:** 10.3389/jpps.2023.11699

**Published:** 2023-10-03

**Authors:** Ye Wang, Zibin Jin, Wenli Xu, Keyu Chen, Lingxin Wei, Dong Yang, Xiaoming Deng, Shiyi Tong

**Affiliations:** Department of Anesthesiology, Plastic Surgery Hospital, Chinese Academy of Medical Sciences and Peking Union Medical College, Beijing, China

**Keywords:** dexmedetomidine, nasal spray, postoperative sleep disturbance, polysomnography, pharmacy

## Abstract

**Purpose:** Dexmedetomidine exerts a sedative effect by promoting the sleep pathway endogenously and producing a state similar to N2 sleep. This study aimed to study the efficacy and safety of dexmedetomidine nasal spray in the treatment of postoperative sleep disturbance.

**Methods:** This study enrolled 120 participants [men and women; age, 18–40 years; American Society of Anesthesiologists grade, I or II] who underwent maxillofacial surgery under general anesthesia through nasotracheal intubation. The participants were randomly divided into three groups: blank control group (BC group), 1.0 μg/kg dexmedetomidine group (1.0 Dex group), and 1.5 μg/kg dexmedetomidine group (1.5 Dex group), with 40 patients allocated to each group. At 21:30 on the night after the operation, the intervention groups were administered their corresponding doses of dexmedetomidine nasal spray. The Pittsburgh Sleep Quality Index (PSQI) scale was used to evaluate the baseline sleep status of participants 1 month preoperatively and on the night after the operation. Polysomnography (PSG) was used to record the sleep status on the night after the operation. We recorded the rescue times of sedative and analgesic drugs on the first night after surgery, adverse reactions, total hospital stay duration, and total costs.

**Results:** Compared with patients in the BC group, those in 1.0 Dex and 1.5 Dex groups had longer N2 sleep duration, were awake for a shorter time after dose administration, woke up less often, and had significantly improved sleep efficiency (*p* < 0.05). Compared with the BC group, the PSQI scores of 1.0 Dex and 1.5 Dex groups were significantly lower on the night after operation, and the proportion of PSQI > 5 was significantly lower (*p* < 0.05). Compared with patients in the BC group and the 1.0 Dex group, those in the 1.5 Dex group had significantly prolonged N3 sleep, reduced frequency of requiring sufentanil rescue, lower incidence of sore throat after surgery, and shorter average length of hospital stay (all, *p* < 0.05).

**Conclusion:** The sleep quality of participants on the night after having undergone maxillofacial surgery was safely and effectively improved by 1.0–1.5 μg/kg dexmedetomidine atomized nasal sprays. Notably, only the latter could prolong N3 sleep. *Level of Evidence II*: Evidence was obtained from at least one properly designed randomized controlled trial.

## Introduction

Postoperative sleep disturbance (POSD) refers to the changes in sleep structure and quality of patients in the early postoperative period. POSD is mainly characterized by decreased rapid eye movement (REM) sleep, increased wake time, and fragmented sleep [1]. During hospitalization, many factors can affect patients’ sleep after operation, such as anxiety, tension, pain, postoperative weakness, medical ward rounds, and noise. Patients’ sleep was disturbed the most on the first night after operation [2]. POSDs can affect patients’ postoperative recovery and adversely affect the aspects of cognition, mood, memory, pain perception, psychomotor function, and metabolic, inflammatory, and immune markers [3]. Improving the sleep quality of hospitalized patients can increase patient comfort and improve surgical outcomes [4]. Dexmedetomidine is a selective alpha-2 adrenergic receptor agonist with sedative, analgesic, and anxiolytic effects [5]. It can effectively alleviate postoperative pain and anxiety and improve the postoperative sleep quality of patients [6]. Dexmedetomidine administration via a nasal spray is simple and convenient and does not irritate the nasal mucosa; furthermore, it is ideal for its higher bioavailability [7]. This mode of administration can avoid the pain and inconvenience associated with venipuncture and intramuscular injection. It has a high degree of patient acceptance and is currently the most commonly used delivery method for this drug clinically [8]. However, there are few clinical studies on the intranasal administration of dexmedetomidine for the treatment of POSD in patients having undergone maxillofacial surgery. The appropriate dose of dexmedetomidine nasal spray for the treatment of sleep disorders requires further validation in clinical trials.

In this double-blind randomized controlled study, different high doses of dexmedetomidine were administered nasally to patients having undergone maxillofacial surgery to compare their effects on the patients’ sleep on the first night after surgery.

### Research hypothesis

Dexmedetomidine nasal spray is safe and effective for alleviating postoperative sleep disturbance in patients undergoing maxillofacial surgery.

## Methods

### Study participants

This study was a single-center double-blind randomized controlled study. The protocol for this trial was approved by the Hospital Ethics Committee of Plastic Surgery Hospital, Chinese Academy of Medical Sciences and Peking Union Medical College (Z2020185). The trial was registered with the China Clinical Trial Registration Center^1^ before patient recruitment (ChiCTR2100041597, Principal investigator: YW, Date of registration: 1 January 2021). The trial was conducted at Plastic Surgery Hospital, Chinese Academy of Medical Sciences and Peking Union Medical College in Beijing, China. All participants were informed of the purpose of this study and provided signed informed consent.

Participants undergoing maxillofacial surgery under general anesthesia and endotracheal intubation at the Hospital, between 2 January 2021 and 27 January 2022 were eligible for this trial. In this study, we included patients who were men or women aged 18–40 years; had an American Society of Anesthesiologists (ASA) physical status of I or II; entered the operating room by 8:00 a.m.; had their endotracheal tube removed within 2 h of operation; were not given patient-controlled analgesia (PCA); and stayed in the post anesthesia care unit (PACU) on the first night after operation. We excluded participants who had a history of other systemic diseases, such as congenital heart disease, hypertension, and epilepsy; who had obstructive sleep apnea–hypopnea syndrome (OSAHS), depression, or were taking sedative and analgesic drugs; who had cysts, tumors, or polyps in their respiratory tract; who had a history of an upper respiratory tract infection in the past 2 weeks, who could not cooperate because of hearing or speech impairment or both, and who refused to enroll.

### Randomization and blinding

In this study, we included 120 participants undergoing maxillofacial plastic surgery. The participants were randomized using a computer-generated random number table^2^ and sealed envelopes and assigned to three groups in a ratio of 1:1:1: blank control group (BC group, *n* = 40), 1.0 µg/kg dexmedetomidine group (1.0 Dex group, *n* = 40), and 1.5 µg/kg dexmedetomidine group (1.5 Dex group, *n* = 40). Treatment allocation was concealed from patients but not from anesthesiologists. The investigators performed intraoperative evaluations and postoperative follow-ups, and participants were blinded to the treatment allocation.

### Anesthesia procedure

After 8 h of fasting, we monitored electrocardiogram (ECG) findings, oxygen saturation (SpO_2_), heart rate (HR), and blood pressure (BP) of the patients. After establishing intravenous access, 0.05 mg/kg midazolam and 0.2 μg/kg sufentanil were administered. Once the patient was sedated, mask ventilation was started, and 10 mg of ephedrine was used to treat the selected nostril; ephedrine could constrict the blood vessels of the nasal mucosa and reduce bleeding during intubation. Thereafter, 2.0 mg/kg propofol and 0.6 mg/kg rocuronium were intravenously injected, followed by continuous mask ventilation for 2 min. Nasotracheal intubation was started after the mandible relaxed. Then, the patients were connected to the anesthesia machine for intermittent barotropic ventilation. Anesthesia was maintained with 7 mg·kg^−1^h^−1^ propofol or 1–2% sevoflurane and 2 µg·kg^−1^min^−1^ remifentanil, with tidal volume (VT) of 8–10 mL/kg, respiratory rate of 12–15 breaths/min, flow rate of 2.5 L/min, and the O_2_: Air ratio of 1.0:1.5 L/min. Intraoperative controlled BP was performed to reduce blood loss.

### Surgical classification

*Single surgery* was defined as undergoing one of the following procedures: mandibular angle and masseter resection, maxillary Lefort I osteotomy, and mandibular sagittal split osteotomy. *Multiple surgeries* were defined as undergoing two or more of the following procedures: maxillary Lefort I osteotomy, mandibular sagittal split osteotomy, mandibular angle/zygomatic/chin osteotomy, and masseter resection.

### Procedures

**FIGURE 1:**
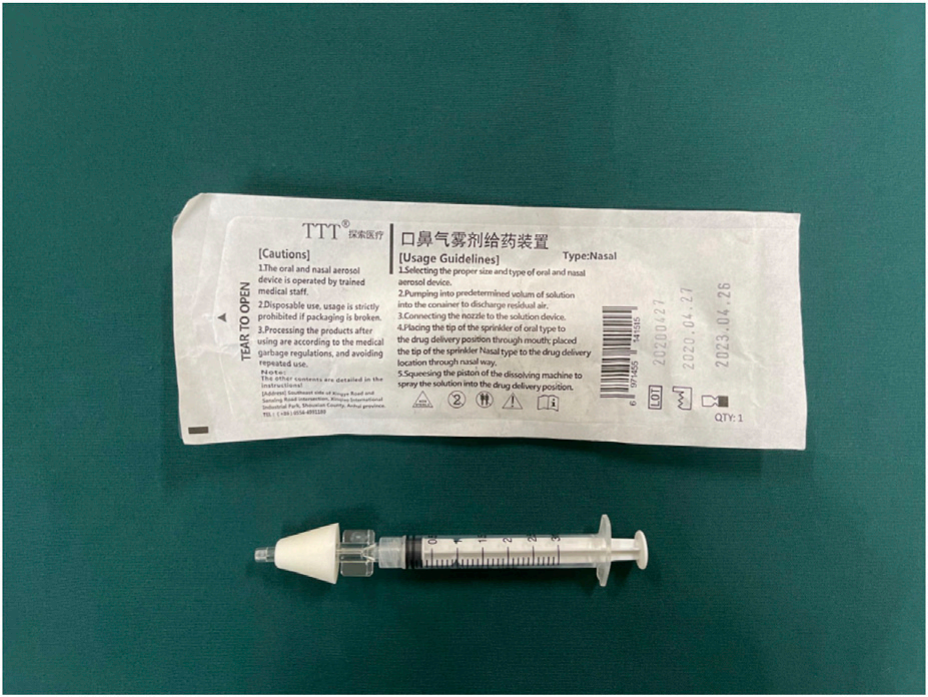
The oral and nasal aerosol device (2 mL*42 mm, Anhui Discovery Medical Device Technology Co., Ltd. China).

Patients in the BC group were not administered any nasal spray. The night after the operation, the 1.0 Dex group was given 1.0 μg/kg dexmedetomidine nasal spray (100 μg/mL, Jiangsu Hengrui Medicine Co., Ltd. Lot number: 210309BP). Similarly, the 1.5 Dex group was given 1.5 μg/kg dexmedetomidine nasal spray. Firstly, the anesthesiologist prepared dexmedetomidine based on patient weight using the oral and nasal aerosol device (2 mL*42 mm, Anhui Discovery Medical Device Technology Co., Ltd. China. Figure 1) and used dexmedetomidine stock solution without dilution. The nurse on duty in the PACU administered the drug at 21:30 on the night of the operation by alternately spraying a small amount of the drug into the left and right nostrils to reduce swallowing. The nurse was not aware of the patient grouping.

**FIGURE 2:**
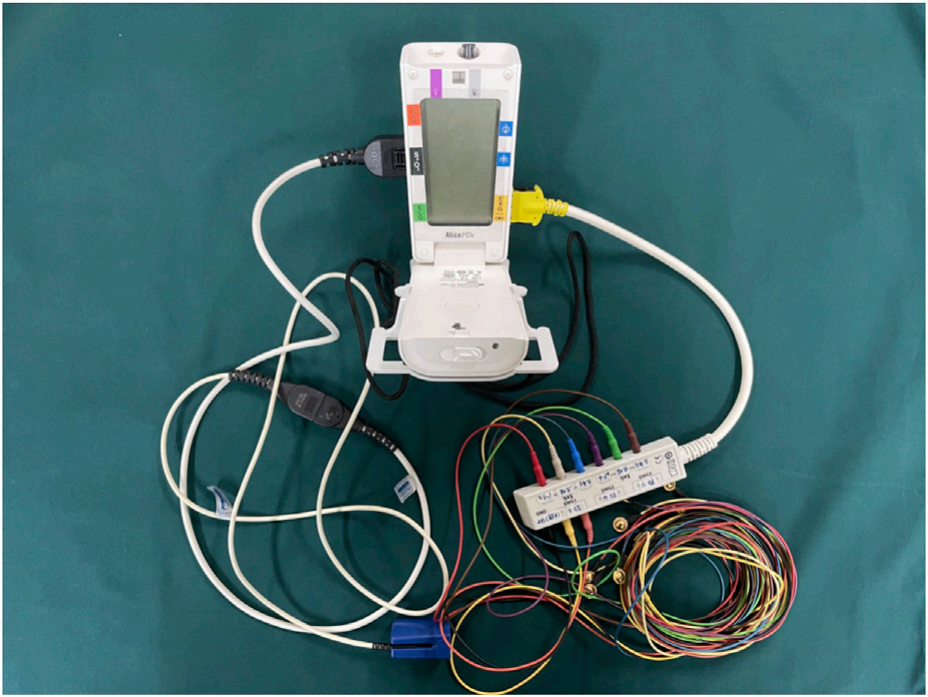
The polysomnography (PSG, Alice PDx, Philips, Respironics Inc. Murrysville, PA, United States).

We used polysomnography (PSG, Alice PDx, Philips, Respironics Inc. Murrysville, PA, United States; Figure 2) to monitor patients’ sleep from 21:30 to 7:00 the next day, and electroencephalogram (EEG), electromyogram (EMG), electrooculogram (EOG), and SpO_2_ findings were recorded. In the night, if the VAS pain score was 4–6 points, the patient was given oral oxycodone/paracetamol (5 mg). If the VAS pain score was 7–10 points, intravenous 0.5 μg/kg sufentanil was administered. If the patient could still not fall asleep after 0:00, 0.02 mg/kg midazolam was intravenously administered. In the event of respiratory depression, the patient was woken up immediately and given oxygen with mask ventilation.

Before premedication, all participants were asked to answer the Pittsburgh sleep quality index (PSQI) questionnaire, which was used to assess their sleeping patterns over the last month (baseline) and the first night after surgery. All participants stayed in the same single room for 1–2 people of the PACU and experienced the same sleeping environment. All questionnaires were scored by the same anesthesiologist with 5 years of experience.

The PSQI questionnaire has seven components (18 items): A, sleep quality; B, sleep latency; C, sleep duration; D, sleep efficiency; E, sleep disorders; F, use of sleep medication; and G, daytime dysfunction.

Each component is scored separately and weighted equally on a scale of 0–3. Thus, total scores range from 0 to 21, with higher scores indicating poorer sleep quality and a score of >5 indicating the presence of a sleep disorder [9]. For questions 5–14 (which discuss the various reasons that could keep patients from falling asleep) and 16–18 (which discuss the frequency of trouble staying awake while driving, eating meals, or engaging in social activity, problems with keeping up the enthusiasm to get things done), the patients could select one of the following options to answer these questions: none, <1 time/week, 1–2 times/week, and ≥3 times/week. The sleep assessment on the night after operation was assessed as none, light, medium, or severe. Item 14 pertained to any specific cause of sleep disturbance that was not covered in items 5–13.

### Primary parameters

PSG report covered the following aspects: sleep stage, frequency and duration of awakening from sleep, sleep efficiency, SPO_2_, and HR. The PSQI scores were also the primary parameters.

### Secondary parameters

The following were the adverse reactions experienced by participants on the night after operation: tachycardia, bradycardia, pain, sore throat, nausea, and vomiting. Other secondary parameters included sedative and analgesic drug rescue times and length and total cost of hospital stay.

### Sample size calculation

After pre-trial dexmedetomidine treatment, the total sample size was calculated based on a previous study that compared the effects of dexmedetomidine (continuous infusion at 0.1 μg kg^−1^ h^−1^; *n* = 31) and placebo (*n* = 30) on postoperative sleep of elderly patients in the ICU. Dexmedetomidine infusion increased the percentage of stage N2 sleep from median 15.8% with placebo to 43.5% with dexmedetomidine (*p* = 0.048) [10]. We assumed that the dexmedetomidine group can prolong N2 phase sleep compared to the blank control group, and there was a statistically significant difference. According to *α* = 0.05, 1−*β* = 0.8, and 10% dropout rate, the total sample size calculated using the Power Analysis and Sample Size software (version 11.0; NCSS, Kaysville, Utah, United States) was about 120, with 40 cases in each group.

### Statistical analysis

All statistical analyses were performed using the Statistical Package for Social Sciences software (version 26.0; SPSS Inc., Chicago, IL, United States). For the three patient groups, demographic features, namely, age, weight, height, and BMI, were presented as means ± standard deviations and ranges, whereas gender, ASA class, and surgical complexity were presented as percentages. PSG sleep staging results are presented as mean ± standard deviation. Sleep patterns were assessed using the PSQI questionnaire, and the scores were calculated. Categorical data are presented as frequencies and percentages, and continuous data are presented as mean ± standard deviation.

The *Shapiro-Wilk* test was used to analyze whether the data were normally distributed. The *ANOVA* was used to compare normally distributed data among the three groups. The least significant difference test was used for *post hoc* pairwise comparisons. The *Kruskal–Wallis H* test was used to compare non-normally distributed data among the three groups. The *χ* ^*2*^ test was used to compare proportions. *p* < 0.05 was considered statistically significant.

## Results

**FIGURE 3:**
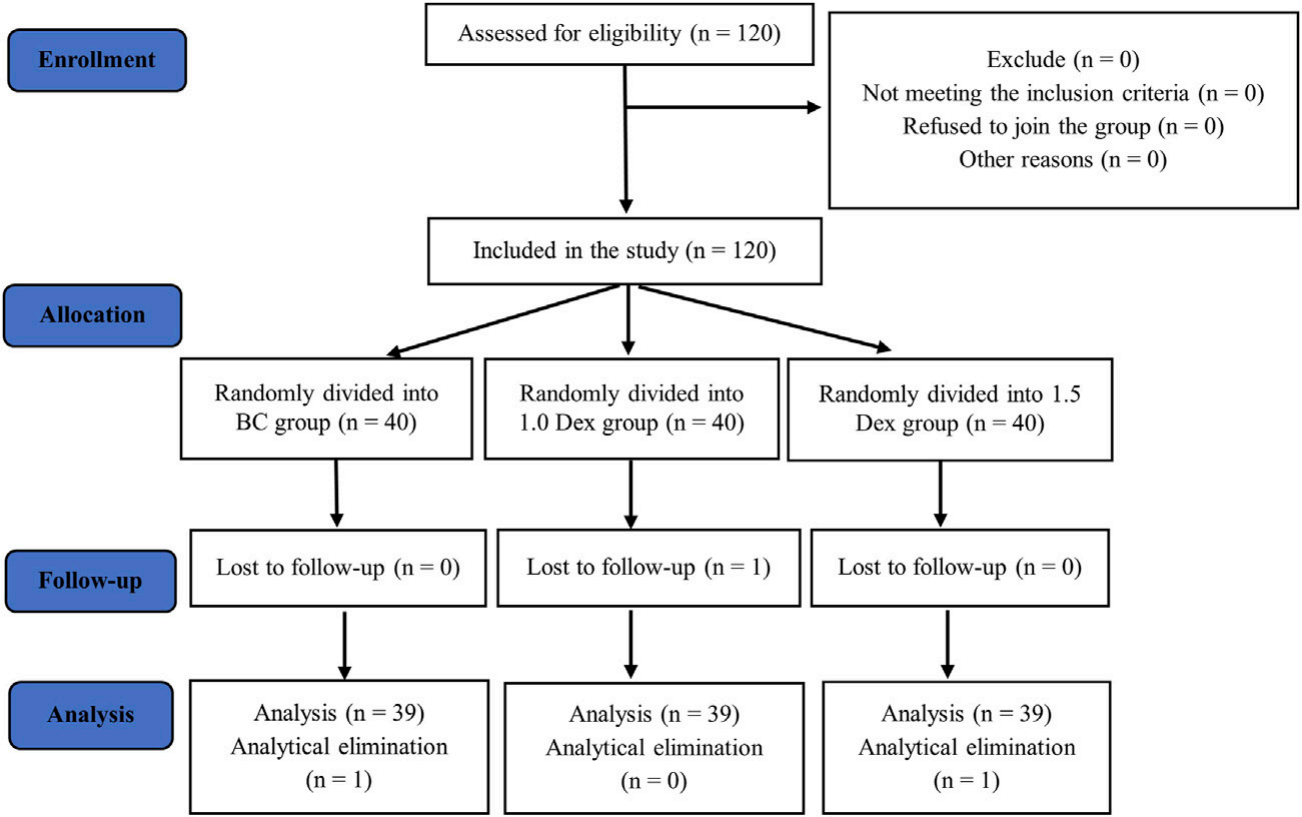
Flow chart.

A total of 120 participants were initially included and randomly divided into three groups. Three participants were excluded because of electrode displacement and loss to follow-up. The final analysis included data collected from 39 patients in each group (Figure 3). The three groups did not significantly differ in terms of continuous variables (age, height, weight, and BMI) or categorical variables (gender, ASA grade, and surgical complexity; Table 1). The three groups did not statistically significantly differ in terms of anesthesia time, operation time, and the major intravenous drugs used during the operation: remifentanil, sufentanil, propofol, and midazolam doses (Table 2).

**TABLE 1:** Participant demographics and surgical complexity.

Variables	BC group (*n* = 39)	1.0 Dex group (*n* = 39)	1.5 Dex group (*n* = 39)	*F*-value	*p*-value
Age (years)	25.7 ± 5.4 [18–36]	25.2 ± 5.0 [18–35]	25.8 ± 4.9	0.137	0.872
Weight (kg)	56.1 ± 13.6 [36–90]	53.4 ± 9.7 [41–85]	51.6 ± 7.3 [41–78]	1.808	0.169
Height (cm)	165.1 ± 8.5 [150.0–184.0]	166.2 ± 8.0 [150.0–190.0]	164.4 ± 6.3 [150–177]	0.573	0.566
BMI (kg/m^2^)	20.4 ± 3.9 [14.4–32.0]	19.2 ± 2.4 [15.2–25.1]	19.1 ± 2.8 [14.7–29.7]	2.017	0.138
Gender (case, %)				*χ* ^*2*^-value	*p*-value
Male	8 (20.5)	4 (10.3)	4 (10.3)	2.317	0.314
Female	31 (79.5)	35 (89.7)	35 (89.7)		
ASA class (case, %)					
ASA I	36 (92.3)	38 (97.4)	39 (100)	3.624	0.163
ASA II	3 (7.7)	1 (2.6)	0 (0)		
Surgical complexity (case, %)					
Single	18 (46.2)	24 (61.5)	15 (38.5)	4.311	0.116
Multiple	21 (53.8)	15 (38.5)	24 (61.5)		

*p* < 0.05 indicates statistically significant difference. *ANOVA* was used to compare age, height, weight, and BMI, and the *χ* ^*2*^ test was used to compare gender, ASA class, and surgical complexity.

**TABLE 2:** Comparison of intraoperative conditions of three groups of participants ( $\bar{x}\pm s$ ).

Variables	BC group (*n* = 39)	1.0 Dex group (*n* = 39)	1.5 Dex group (*n* = 39)	*F*-value	*p*-value
Anesthesia time (min)	323.7 ± 113.8	292.6 ± 88.7	279.7 ± 72.5	2.867	0.238
Operation time (min)	266.3 ± 99.8	222.6 ± 87.7	231.9 ± 56.7	3.845	0.146
Sufentanil (μg)	25.3 ± 7.8	23.0 ± 6.8	24.1 ± 6.2	2.109	0.348
Remifentanil (μg)	1371.9 ± 655.6	1230.8 ± 450.9	1114.5 ± 345.5	2.764	0.251
Propofol (mg)	510.5 ± 425.7	595.9 ± 551.8	375.0 ± 324.0	1.833	0.400
Midazolam (mg)	1.5 ± 1.5	1.4 ± 1.1	1.7 ± 1.4	0.534	0.766

*p* < 0.05 indicates statistically significant difference. The *Kruskal–Wallis H* test was used to analyze the time of anesthesia, the time of operation, and the main intravenous drugs used in the operation among the three groups.

### Primary parameters

#### PSG reports

Compared with the BC group, the 1.0 Dex group and the 1.5 Dex group had prolonged N2 sleep (BC, 1.0 Dex, 1.5 Dex: 181.4 ± 72.2, 240.7 ± 89.4, 259.4 ± 71.5 min), shorter wake-up time (BC, 1.0 Dex, 1.5 Dex: 187.9 ± 101.0, 91.4 ± 67.0, 97.4 ± 75.3 min), fewer awakenings from sleep (BC, 1.0 Dex, 1.5 Dex: 31.1 ± 15.8, 19.8 ± 10.7, 17.6 ± 11.0 times), and significantly improved sleep efficiency (BC, 1.0 Dex, 1.5 Dex: 67.1 ± 17.7, 84.0 ± 11.8, 82.7 ± 13.0%). However, these aspects did not significantly differ between 1.0 Dex and 1.5 Dex groups. Compared with the BC group and the 1.0 Dex group, the 1.5 Dex group was associated with significantly prolonged N3 sleep (BC, 1.0 Dex, 1.5 Dex: 42.8 ± 38.4, 53.6 ± 74.7, 75.0 ± 65.7 min). The three groups did not statistically significantly differ in terms of N1 sleep and REM sleep stages, average HR during sleep, highest HR during sleep, and lowest blood oxygen saturation during sleep (Table 3).

**TABLE 3:** Comparison of sleep rhythms in the three groups of participants on the night after operation ( $\bar{x}\pm s$ ).

Variables	BC group (*n* = 39)	1.0 Dex group (*n* = 39)	1.5 Dex group (*n* = 39)	*p*-value
N1 (min)	98.9 ± 45.9	99.4 ± 55.6	90.2 ± 57.9	0.536
N2 (min)	181.4 ± 72.2	240.7 ± 89.4^a^	259.4 ± 71.5^a^	**<0.0001**
N3 (min)	42.8 ± 38.4	53.6 ± 74.7	75.0 ± 65.7^a^ ^,^ ^b^	**0.029**
REM (min)	62.2 ± 70.4	85.0 ± 101.3	48.1 ± 57.4	0.265
Wake time (min)	187.9 ± 101.0	91.4 ± 67.0^a^	97.4 ± 75.3^a^	**<0.0001**
Awakening times (times)	31.1 ± 15.8	19.8 ± 10.7^a^	17.6 ± 11.0^a^	**<0.0001**
Sleep efficiency (%)	67.1 ± 17.7	84.0 ± 11.8^a^	82.7 ± 13.0^a^	**<0.0001**
Sleep average heart rate (beats per min)	83.9 ± 13.5	77.1 ± 9.0	76.5 ± 12.3	0.051
Sleep peak heart rate (beats per min)	113.4 ± 13.4	110.2 ± 15.9	110.2 ± 12.6	0.402
Sleep minimum SpO_2_ (%)	90.0 ± 4.4	91.2 ± 3.1	90.0 ± 3.7	0.200

*p* < 0.05 indicates statistically significant difference. For non-normally distributed data, the *Kruskal–Wallis H* test was used for between-group comparisons and pairwise comparisons. Bold: *p*-value < 0.05.

^a^ There is a statistical difference compared with the BC group.

^b^ There is a statistical difference compared with the 1.0 Dex group.

#### PSQI score

**FIGURE 4:**
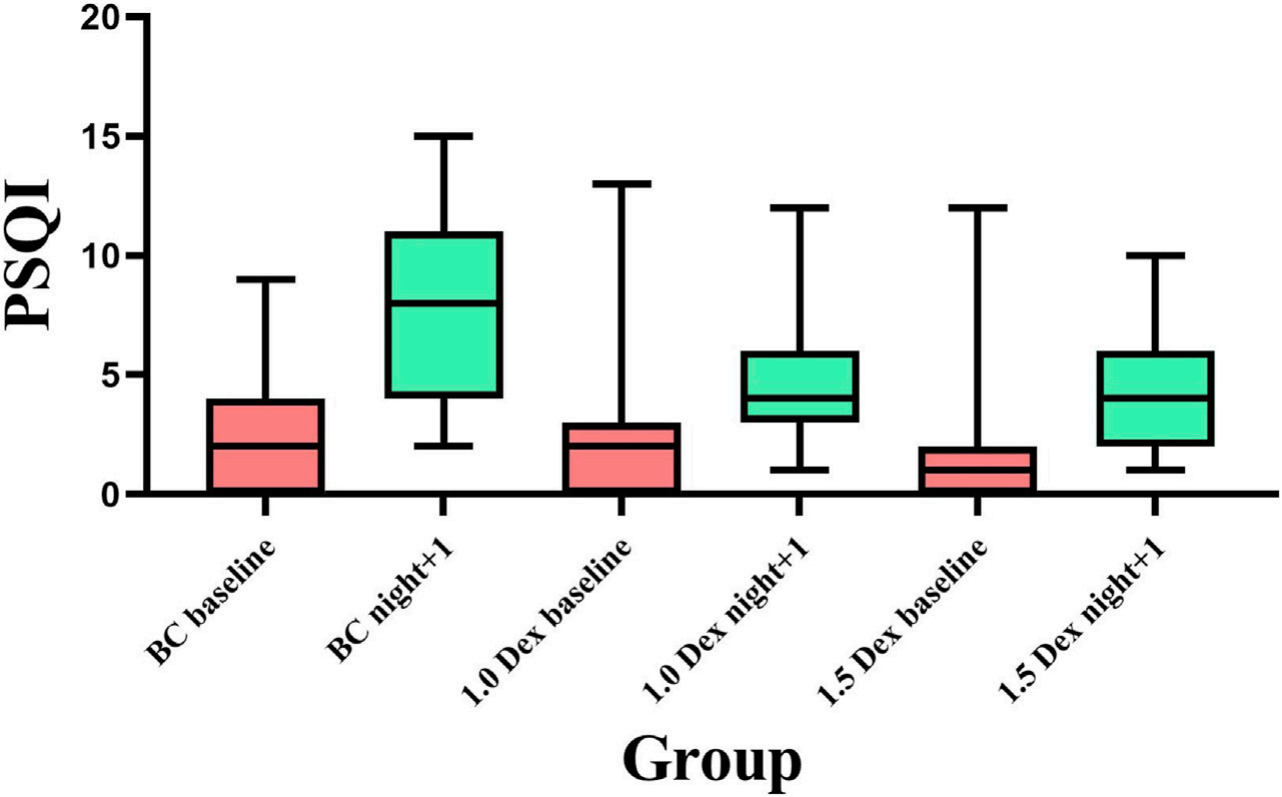
Comparison of PSQI scores among the three groups.

The baseline PSQI scores did not statistically significantly differ among the three groups. Compared with the baseline, the PSQI scores of the three groups were significantly increased on the night after the operation, and the proportion of PSQI > 5 was significantly increased as well. Compared with the BC group, the PSQI scores of 1.0 Dex and 1.5 Dex groups were significantly reduced on the night after the operation (BC, 1.0 Dex, 1.5 Dex:7.8 ± 3.6, 4.9 ± 2.8, 4.1 ± 2.0), and the proportion of PSQI > 5 was also significantly reduced (BC, 1.0 Dex, 1.5 Dex:71.8, 25.6, 25.6%). However, there was no statistically significant difference between 1.0 Dex and 1.5 Dex groups (Table 4 and Figure 4).

**TABLE 4:** Comparison of PSQI scores of three groups.

Variables		BC group (*n* = 39)	1.0 Dex group (*n* = 39)	1.5 Dex group (*n* = 39)	*p*-value for intergroup comparisons
A: Sleep quality score	Baseline	0.6 ± 0.8 [0–3]	0.4 ± 0.7 [0–3]	0.4 ± 0.7 [0–2]	0.449
	The night after operation	2.1 ± 0.8 [1–3]	1.6 ± 0.9 [0–3]^a^	1.6 ± 0.8 [0–3]^a^	**0.041**
	*p*-value for intragroup comparison	**<0.0001**	**<0.0001**	**<0.0001**	
B: Sleep latency score	Baseline	0.7 ± 1.0 [0–3]	0.5 ± 0.9 [0–3]	0.3 ± 0.7 [0–3]	0.072
	The night after operation	2.1 ± 0.9 [0–3]	1.4 ± 0.8 [0–3]^a^	0.4 ± 0.6 [0–3]^a^ ^,^ ^b^	**<0.0001**
	*p*-value for intragroup comparison	**<0.0001**	**<0.0001**	0.448	
C: Sleep duration score	Baseline	0.3 ± 0.7 [0–3]	0.2 ± 0.6 [0–2]	0.2 ± 0.5 [0–2]	0.621
	The night after operation	0.9 ± 1.1 [0–3]	0.2 ± 0.7 [0–3]^a^	0.0 ± 0.2 [0–1]^a^	**<0.0001**
	*p*-value for intragroup comparison	**0.008**	0.855	**0.034**	
D: Sleep efficiency score	Baseline	0.0 ± 0.0 [0–0]	0.1 ± 0.2 [0–1]	0.0 ± 0.2 [0–1]	0.361
	The night after operation	1.1 ± 1.1	0.3 ± 0.7 [0–3]^a^	0.5 ± 0.3 [0–2]^a^	**<0.0001**
	*p*-value for intragroup comparison	**<0.0001**	**0.024**	0.655	
E: Sleep disorders score	Baseline	0.4 ± 0.5 [0–1]	0.5 ± 0.5 [0–1]	0.5 ± 0.5 [0–1]	0.903
	The night after operation	1.0 ± 0.2 [1–2]	1.0 ± 0.2 [0–1]	1.0 ± 0.0 [1–1]	0.226
	*p*-value for intragroup comparison	**<0.0001**	**<0.0001**	**<0.0001**	
F: Use of sleep medication score	Baseline	0.0 ± 0.0 [0–0]	0.1 ± 0.2 [0–1]	0.0 ± 0.2 [0–1]	0.361
	The night after operation	0.4 ± 0.5 [0–1]	0.3 ± 0.5 [0–1]	0.5 ± 0.5 [0–1]	0.115
	*p*-value for intragroup comparison	**<0.0001**	**0.013**	**<0.0001**	
G: Daytime dysfunction score	Baseline	0.6 ± 0.7 [0–3]	0.5 ± 0.7 [0–3]	0.2 ± 0.7 [0–3]^a^	**0.005**
	The night after operation	0.3 ± 0.5 [0–1]	0.2 ± 0.5 [0–2]	0.5 ± 0.6 [0–2]	0.085
	*p*-value for intragroup comparison	**0.034**	**0.033**	**0.020**	
PSQI score	Baseline	2.7 ± 2.8 [0–9]	2.2 ± 2.6 [0–13]	1.6 ± 2.4 [0–12]	0.210
	The night after operation	7.8 ± 3.6 [2–15]	4.9 ± 2.8 [1–12]^a^	4.1 ± 2.0 [1–10]^a^	**<0.0001**
	*p*-value for intragroup comparison	**<0.0001**	**<0.0001**	**<0.0001**	
PSQI score > 5 [case (%)]	Baseline	7 (17.9)	3 (7.7)	2 (5.1)	0.142
	The night after operation	28 (71.8)	10 (25.6)^a^	10 (25.6)^a^	**<0.0001**
	*p*-value for intragroup comparison	**<0.0001**	**0.020**	**0.005**	

*p* < 0.05 indicates statistically significant difference. PSQI scores were non-normally distributed, and the *Kruskal–Wallis H* test was used for between-group comparisons and pairwise comparisons. Bold: *p*-value < 0.05.

^a^ There is a statistical difference compared with the BC group.

^b^ There is a statistical difference compared with the 1.0 Dex group.

The baseline scores of the aforementioned A-F items did not significantly differ. Regarding scores for sleep quality (item A), sleep duration (item C), and sleep efficiency (item D), the postoperative scores of the three groups were significantly increased compared with the baseline score. Postoperative scores of items A, C, and D decreased significantly in the 1.5 Dex group, but there was no statistical difference between the 1.0 Dex group and the 1.5 Dex group. Regarding the sleep latency score (item B), the postoperative sleep latency scores of the BC group and the 1.0 Dex group were significantly increased compared with the baseline score, and the 1.5 Dex group had the least score. Regarding the scores for sleep disorders (E) and use of sleep medication (F), the scores on the night after operation in all three groups were significantly increased compared with the baseline score; however, there was no statistically significant difference in these scores among the three groups. Regarding the score for daytime dysfunction (item G), the 1.5 Dex group had a lower baseline score than the BC group. The scores of the BC group and the 1.0 Dex group were decreased and that of the 1.5 Dex group was increased compared with the baseline score; however, the scores of the three groups did not statistically significantly differ on the night after operation (Table 4).

### Secondary parameters

In terms of the supplementation of sedative and analgesic drugs on the night after operation, the three groups did not statistically significantly differ in terms of the administered doses of paracetamol, oxycodone, and midazolam. Sufentanil rescue was needed fewer times in the 1.5 Dex (10.3%) group than in the BC group (33%) (Table 5).

**TABLE 5:** Comparison of sedative and analgesic drug recovery among the three groups on the night after operation [cases (%)].

Variables	BC group (*n* = 39)	1.0 Dex group (*n* = 39)	1.5 Dex group (*n* = 39)	*p*-value
Sufentanil	13 (33.3)	5 (12.8)	4 (10.3)^a^	**0.017**
Paracetamol oxycodone	2 (5.1)	3 (7.7)	6 (15.4)	0.271
Midazolam	2 (5.1)	2 (5.1)	2 (5.1)	1.0

*p* < 0.05 was considered statistically significant, and the *χ* ^*2*^ test was used for proportional analysis. Bold: *p*-value < 0.05.

^a^ There is a statistical difference compared with the BC group.

Regarding the adverse reactions noted on the night after operation, the incidences of bradycardia, tachykinesia, easy or early awakening, surgical wound pain, nightmares, and nausea and vomiting did not significantly differ among the three groups. The incidence of sore throat was lower in the 1.5 Dex group (10.3%) than in the 1.0 Dex group (33.3%). The total cost of hospitalization did not significantly differ among the three groups; however. the length of hospital stay was significantly lower in the 1.5 Dex group (5.4 ± 1.6 days) than in the BC group (7.1 ± 2.5 days) (Table 6).

**TABLE 6:** The occurrence of adverse reactions in the three groups on the night after operation [cases (%)], hospitalization time, and total hospitalization expenses.

Variables	BC group (*n* = 39)	1.0 Dex group (*n* = 39)	1.5 Dex group (*n* = 39)	*p*-value
Bradycardia	8 (20.5)	14 (35.9)	18 (46.2)	0.056
Tachycardia	31 (79.5)	27 (69.2)	25 (64.1)	0.313
Easy to wake up or wake up early	34 (87.2)	36 (92.3)	37 (94.9)	0.465
Surgical wound pain	25 (64.1)	24 (61.5)	22 (56.4)	0.778
Nightmare	2 (5.1)	1 (2.6)	1 (2.6)	0.772
Nausea and vomiting	3 (7.7)	2 (5.1)	3 (7.7)	0.874
Sore throat	10 (25.6)	13 (33.3)	4 (10.3)^b^	**0.048**
Hospital stay (days)	7.1 ± 2.5	6.5 ± 2.0	5.4 ± 1.6^a^	**0.003**
Total hospitalization expenses (Chinese Yuan)	55059.8 ± 16782.5	49937.1 ± 13582.0	57983.8 ± 17067.6	0.081

*p* < 0.05 was considered statistically significant. The *Wilcoxon signed-rank* test was used to analyze data with a skewed distribution, and the *χ* ^*2*^ test was used for proportion analysis. Bold: *p*-value < 0.05.

^a^ There is a statistical difference compared with the BC group.

^b^ There is a statistical difference compared with the 1.0 Dex group.

## Discussion

Maxillofacial plastic surgery changes the contour of the face by modifying the bone structure of the maxillofacial region. The procedure is long and traumatic and requires general anesthesia and controlled BP reduction to reduce blood loss during the operation. Postoperative pressure bandaging is required, and there is excessive oral secretion [11, 12]. Patients having undergone maxillofacial plastic surgery are extremely uncomfortable and have high levels of anxiety on the night after operation; these issues are severely detrimental to their sleep. If these issues remain unresolved, they can affect the prognosis and cognitive function of the patients, exacerbate postoperative pain, and even induce cardiovascular events [13]. Dexmedetomidine exerts its hypnotic action through activation of central pre- and postsynaptic α_2_ -receptors in the locus coeruleus, thereby inducting a state of unconsciousness similar to natural sleep, with the unique aspect that patients remain easily rousable and cooperative [14].

In this study, 1.0 and 1.5 μg/kg dexmedetomidine stock solution atomized nasal sprays were used to treat sleep disturbance on the night after operation in participants having undergone maxillofacial surgery. The results showed that dexmedetomidine nasal sprays at both concentrations could effectively prolong N2 sleep (BC, 1.0 Dex, 1.5 Dex: 181.4 ± 72.2, 240.7 ± 89.4, 259.4 ± 71.5 min), shorten the waking time, reduce the number of awakenings from sleep, significantly improve sleep efficiency, reduce PSQI score, and reduce the incidence of sleep disorders. Notably, 1.5 μg/kg dexmedetomidine nasal spray could also effectively prolong N3 sleep. In addition, 1.5 μg/kg dexmedetomidine nasal spray could also reduce the number of times sufentanil rescue had to be used postoperatively; furthermore, it reduced the incidence of postoperative sore throat and the length of hospital stay. Wu XH, et al. founded that dexmedetomidine infusion increased the percentage of stage N2 sleep from 15.8% with placebo to 43.5% with dexmedetomidine; it also prolonged the total sleep time, decreased the percentage of stage N1 sleep, increased the sleep efficiency, and improved the subjective sleep quality. Dexmedetomidine increased the incidence of hypotension without significant intervention [10]. Although the administration methods were different, the results were consistent with our study.

Sleep includes REM sleep and non-rapid eye movement (NREM) sleep. NREM sleep is subdivided into N1, N2, and N3 sleep, representing progressively deeper stages of sleep. In this study, using PSG, we could objectively analyze sleep staging by studying EEG, EMG, and electrooculography findings [15]. Unlike other sedative drugs, dexmedetomidine exerts a sedative effect by promoting an endogenous sleep pathway and producing a state similar to N2 sleep [16]. Xu et al. [17] showed that intravenous infusion of dexmedetomidine (average dose, 104.60 μg ± 27.93 μg) can induce N2 sleep with a similar proportion to natural sleep. Chamadia et al. [18] confirmed that oral dexmedetomidine solid capsules at night promote N2 sleep. The results of the present study revealed that dexmedetomidine nasal spray effectively prolongs N2 sleep. Increasing the dose to 1.5 μg/kg could also prolong N3 sleep.

Dexmedetomidine is convenient to administer via a nasal spray, which supports rapid onset of action. Intranasal bioavailability was estimated to be 40.6% and 40.7% for atomisation and drops respectively. Degree and duration of sedation were similar for i.v. and intranasal administration [7]. Following intranasal administration, peak plasma concentrations of dexmedetomidine were reached in 38 min and its absolute bioavailability was 65% [19]. Yoo et al found that intranasal bioavailability was 82% [8]. Intranasal route has onset of action in 45 min with peak effect in 90–100 min. There is no difference in the pharmacokinetic profile of either males or females, and both have similar protein binding [14]. Our findings also confirmed that 1.5 μg/kg dexmedetomidine when administered via a nasal spray not only improves the sleep quality but also reduces the frequency of requiring sufentanil rescue, the incidence of postoperative sore throat, and the length of hospital stay. Dexmedetomidine administered via a nasal spray is a non-invasive, safe, and effective method for the treatment of postoperative sleep disorders with a wide range of applications; for example, it reduces pain and improves sleep quality after nasal endoscopic surgery and works as an effective sedative agent for pediatric examination [20–22]. The protocol of administering 3  μg/kg dexmedetomidine injection combined with 0.3 mg/kg midazolam nasal drops has been reported to be safe, easy to use, and highly successful in pediatric patients when administered before their craniocerebral magnetic resonance imaging examination [22]. Xu et al. [23] reported that the effective dose of dexmedetomidine nasal spray to induce sleep in 3–6 years-old children was 1.76 μg/kg. The majority of anesthesiologists use dexmedetomidine in pediatrics for premedication and procedural sedation and in the ICU. The dosage varied widely and ranged from 0.2 to 5 μg/kg for nasal premedication and 0.2 to 8 μg/kg for nasal procedural sedation [24]. In the present study, the use of 1.0–1.5 μg/kg dexmedetomidine nasal spray on the night after maxillofacial plastic surgery could not only improve the postoperative sleep quality of the participants but also ensure unobstructed airway in the participants. The doses were safe and effective. Using the optimized nasal spray method can greatly improve the bioavailability of the test drug in healthy adults [25]. Intranasal administration of 1.0 μg/kg dexmedetomidine is reportedly more effective than buccal administration of 1.0 μg/kg dexmedetomidine for premedication in children [26]. Intranasal dexmedetomidine is a superior sedative to administer before performing electroencephalograms in children with autistic spectrum disorders [27]. Dexmedetomidine effectively induces sleep when administered via a nasal spray, and continuous low-dose intravenous infusion is effective for maintaining sleep [28]. Dexmedetomidine is now being used as part of ERAS protocols to create a satisfactory postoperative outcome with reduced opioid consumption in the PACU [29].

This study selected patients who underwent maxillofacial surgery in the morning. When dexmedetomidine was postoperatively administered via a nasal spray, the anesthesia withdrawal time was more than 4 h. After the operation, additional sedative and analgesic drugs were used according to the patient’s voluntary requirements. A common complication of dexmedetomidine is slow HR. In this study, bradycardia occurred in 8, 14, and 18 patients in the BC group, 1.0 μg/kg Dex group, and 1.5 μg/kg Dex group on the night after the operation, respectively; however, the HR did not reduce beyond 50 beats/min in any of the patients, related to sleep state. Although the HR decreased during sleep, no heart rate-increasing medication was administered.

## Limitations

The study did not observe the prognosis of patients and the longer term impact of dexmedetomidine nasal spray on postoperative sleep disorders. Inconsistent surgical methods may have different effects on postoperative pain and sleep of patients. The PSG polysomnography did not collect data on respiratory events. This study did not compare the effects of different drug administration methods, such as continuous intravenous infusion, sublingual administration, and nasal drip, on sleep in patients undergoing maxillofacial surgery. Furthermore, this study did not compare the effect of atomized nasal spray of dexmedetomidine with that of other sedatives.

## Conclusion

Notably, 1.0–1.5 μg/kg dexmedetomidine administered via a nasal spray on the night after operation can safely and effectively prolong N2 sleep and shorten wake-up time in participants having undergone maxillofacial plastic surgery on the night after operation. Furthermore, it was associated with fewer awakenings from sleep, significantly improved sleep efficiency, and reduced incidence of sleep disorders. Interestingly, 1.5 μg/kg dexmedetomidine could also prolong N3 sleep, reduce the number of times sufentanil rescue had to be used postoperatively, reduce the incidence of postoperative sore throat, and reduce the length of hospital stay.

## Author’s note

Our department had obtained the permission of PSQI and translated it into Chinese for the understanding of Chinese participants.

## Data Availability

The raw data supporting the conclusion of this article will be made available by the authors, without undue reservation.

## Footnotes

1
http://www.chictr.org.cn

2
http://www.randomization.com
